# How do COPD patients respond to exacerbations?

**DOI:** 10.1186/1471-2466-11-43

**Published:** 2011-08-19

**Authors:** Jaap CA Trappenburg, David Schaap, Evelyn M Monninkhof, Jean Bourbeau, Gerdien H de Weert-van Oene, Theo JM Verheij, Jan-Willem J Lammers, Augustinus JP Schrijvers

**Affiliations:** 1 Julius Center for Health Sciences and Primary Care, University Medical Center Utrecht, Heidelberglaan 100, 3584 CX Utrecht, The Netherlands; 2 Respiratory Epidemiology and Clinical Research Unit, Montreal Chest Institute, McGill University Health Center, McGill University, 3650 St. Urbain Street, Montreal, Canada; 3 Department of Respiratory Medicine, University Medical Center Utrecht, Heidelberglaan 100, 3584 CX Utrecht, The Netherlands

## Abstract

**Background:**

Although timely treatment of COPD exacerbations seems clinically important, nearly half of these exacerbations remain unreported and subsequently untreated. Recent studies have investigated incidence and impact of failure to seek medical treatment during exacerbations. Yet, little is known about type and timing of other self-management actions in periods of symptom deterioration. The current prospective study aims at determining the relative incidence, timing and determinants of three types of patient responses.

**Methods:**

In a multicentre observational study, 121 patients (age 67 ± 11 years, FEV_1_pred. 48 ± 19) were followed for 6 weeks by daily diary symptom recording. Three types of action were assessed daily: planning periods of rest, breathing techniques and/or sputum clearing (type-A), increased bronchodilator use (type-B) and contacting a healthcare provider (type-C).

**Results:**

Type-A action was taken in 70.7%, type-B in 62.7% and type C in 17.3% of exacerbations (n = 75). Smokers were less likely to take type-A and B actions. Type-C actions were associated with more severe airflow limitation and increased number of hospital admissions in the last year.

**Conclusions:**

Our study shows that most patients are willing to take timely self-management actions during exacerbations. Future research is needed to determine whether the low incidence of contacting a healthcare provider is due to a lack of self-management or healthcare accessibility.

## Background

Chronic obstructive pulmonary disease (COPD) is characterised by a progressive decline in respiratory function, exercise capacity and health status [1]. This underlying disease state is interrupted by episodes of acute worsening in respiratory symptoms. If these deteriorations are beyond individual day-to-day variability, these are defined as exacerbations [2]. It is widely recognized that acute exacerbations play a central role in COPD-related morbidity and mortality [1]. Exacerbations are associated with marked physiologic deterioration that may affect disease progression by accelerating reductions in forced expiratory volume in 1 s (FEV_1_) [3,4], have a significant negative effect on the individual's health-related quality of life (HRQoL) [5,6] and generate an increasing burden on health services and economic costs [7]. Several studies have shown that almost 50% of exacerbations remain unreported and subsequently do not receive adequate treatment [8-10]. Although unreported exacerbations are often considered to be mild, recent studies have shown that these exacerbations may have short and long term consequences on patients health-related quality of life [9,10]. Moreover, early treatment and thus early recognition of exacerbation symptoms has shown to improve outcome of exacerbations [11]. In addition, patients who refrain from seeking treatment during these episodes or have less self-management capacity show higher hospitalization rates compared to those who seek early treatment from physicians or have better self-management capacity [12].

Until now, little is known why exacerbations remain unreported except that they seem shorter in length, display less shortness of breath and are less associated with cough [9,13]. To improve the understanding on delay and failure to seek medical treatment, more knowledge is needed on exacerbation perception and health behaviour by patients specifically during exacerbation episodes. This knowledge can give a more profound direction in approaches aiming at increasing exacerbation related self-management, such as individualized action plans [14].

The current observational study aims at a prospective evaluation of the hypothesis that whilst not contacting a healthcare provider in the event of an exacerbation, patients might take other types of self-management action. More specifically, the objectives of the study were: (1) to determine the relative incidence of different types of action taken by patients in the event of an exacerbation, (2) to determine the time patients take to act in the event of an exacerbation and (3) to compare characteristics of patients according to those who take or do not take timely action measures.

## Methods

### Patients

Between January and March 2008, COPD patients were recruited from inpatient (post-discharge) and outpatient clinics from the University Medical Hospital in Utrecht, 6 peripheral hospitals and 5 general practices. Patients were followed during a period of 6 weeks (42 days). Patients required the following criteria (extracted from chart review): 1) aged over 40 years; 2) primarily diagnosis of COPD; 3) complaints of dyspnea and/or chronic cough with or without hypersecretion; 4) history of smoking (> 20 years of smoking or > 15 packyears); 5) post-bronchodilator FEV/FVC of ≤ 0,7 according to the global initiative for chronic obstructive lung disease (GOLD) standards [1]. Patients were excluded if they had a primary diagnosis of asthma, cardiac disease or any medical condition other than COPD with functional limitation. Ethical approval was obtained from the Medical-Ethical Review Committee (METC) of the UMC Utrecht, and all patients gave their written informed consent prior to inclusion.

### Study Design

This is an observational study with a prospective evaluation of patient daily decision in the event of an exacerbation. Data were obtained from a pilot-study aiming at the development of an Action Plan for COPD patients. At recruitment, patients were asked to record respiratory symptom deteriorations on a daily basis, using a diary card [15]. Patients were instructed to fill in this diary at a fixed moment of the day; i.e. after evening diner. The diary card consisted of major symptoms (dyspnea, sputum volume and sputum color), and minor symptoms (sore throat, fever, cough, common cold, wheezing). Major symptoms were scored when an increase was perceived. Minor symptoms were scored when they were present that day, and not part of the patients' normal symptom status. In addition, patients were instructed to note whether they contacted a healthcare provider, increased their inhalation medication, started a course of corticosteroids or antibiotics or increased their attention on planning periods of rest, breathing techniques and sputum expectoration. Patients were contacted by the investigators by telephone after the first 7 days to review their compliance and understanding with regard to the daily assessments. Patients did not receive any additional written or verbal self-management instructions on how to act in the event of an exacerbation.

After completion of the study period, patients' records were examined for baseline and healthcare utilization data (visits to the physician, the emergency department and hospital from the medical records). Self-reported healthcare contacts were matched with contacts extracted from the medical records and subsequently the exact date was ascertained. Charlson's comorbidity index (comorbidities extracted from chart review) was used to determine the degree of comorbidity [16]. This index is based on relative risks of mortality, in which 19 conditions are assigned with values of 1, 2, 3, or 6 (all other conditions are given a score of 0). The weights were then summed for each patient.

#### Exacerbations

A symptom-based exacerbation was confirmed if, for at least two consecutive days, patients experienced a worsening of at least one of three major symptoms (increased sputum amount, changed sputum color/purulence, and increased dyspnea) [9,10,17]. Severity of exacerbation was divided in type 1, 2 and 3, with addition of a type 4, by definition of Vijayasaratha et al. [18]. A type 1 exacerbation included an increase in three major symptoms, a type 2 exacerbation was defined as an increase in two major symptoms, a type 3 exacerbation was defined as an increase in one major and at least one minor symptom, and a type 4 exacerbation when only 1 major symptom was increased without the addition of any minor symptoms. Daily symptoms were binary coded and summed to give a daily symptom count. Major symptoms are scored as: normal = 0; small increase = 1; or clear increase = 2. The minor symptoms were scored 0 and 1, respectively. The sum of these scores resulted in a daily symptom count ranging from 0-11 points. Exacerbation onset was taken as the first day on which these symptom criteria were met. As a second estimation of exacerbation severity, the number of individual increased symptoms was counted up in a minor, major and total symptom count. All first exacerbations were taken into account, provided that at least three consecutive major symptom free days were counted before exacerbation onset. Exacerbation recovery was regarded as the first of three consecutive major symptom free days (back to baseline or new level of stability).

#### Symptomatic days

Symptomatic days were defined as separated days with increased minor or major symptoms, preceded by at least three consecutive days without symptom change. This operational definition was to insure that these days were not part of an exacerbation episode.

#### Measurement of actions taken by patients

Three types of action measures were evaluated. Selection of these actions was made according to a large multinational interview-based study in which patients were asked to retrospectively report on exacerbation experiences [19]. These actions were operationally defined as types: A) increased attention on planning periods of rest, breathing techniques and/or sputum expectoration; B) increasing the use of inhaled medication from the regular dial in the normal dose of inhalation medication. Type C actions were defined as contacting (telephone consultation or visit) a healthcare provider.

### Statistical analysis

All patients that returned their diaries were included in analysis. Statistical analysis was performed using SPSS version 14.01. Normally distributed data are presented as mean (SD) and otherwise as median (interquartile range; IQR). Incidence and timing of the three different types of actions were evaluated between three days before to ten days after exacerbation onset. To compare characteristics of patients who take or do not take timely action measures, differences in patient, event and system characteristics were tested by Chi-squared test, Mann-Whitney U-test or Student t-test depending on the level of measurement. A p-value of < 0.05 was considered to be statistically significant.

## Results

**Figure 1:**
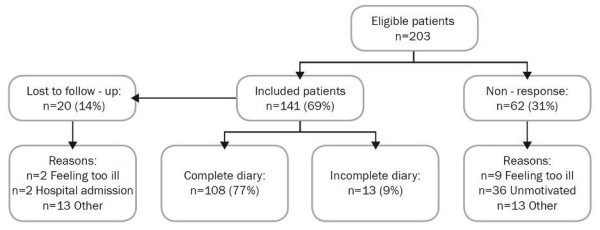
**Flow chart of participants through the study**.

A total of 203 COPD patients were asked to participate in the study (Figure 1). Of these, a total number of 141 (69%) patients were enrolled in the study of which 20 (14%) were lost to follow up, leaving 121 patients (86%) eligible for analysis. Patients lost to-follow did not significantly differ in terms of baseline characteristics. Thirteen patients (9%) returned an incompletely filled in diary. To prevent selective loss to follow up, these diaries were included in the analysis.

Table 1 shows the baseline characteristics of the included patients. The patients were recruited from scheduled outpatient clinic visits (68.6%), scheduled general physician visits (11.6%) and patients at discharge after hospital admission for treatment of an exacerbation (19.8%). About 60% of the patients were male and the average age was 67 years. Patient had on average moderate to severe COPD (FEV_1_%pred: 47.7 ± 18.5) and 23.1% were smoking at the time of enrolment. Comorbidity scored according to the Charlson's index was low. The 121 diaries contained 5082 diary cards, of which 238 (5%) were missing. The number of missing diary days ranged from 4 to 32 days. The mean total time spent in the study was 40.03 (± 5.74) days.

**Table 1:** Baseline characteristics of all patients and patients with and without at least one symptom based exacerbations

Characteristics	All patients	No symptom-based exacerbation	At least one symptom-based exacerbation
**Number of patients**	121	46	75
**Sex, male**	74 (61.2)	27 (58.7)	47 (62.7)
**Age, yr**	67.4 ± 10.5	68.6 ± 11.3	66.6 ± 9.9
**FEV_1_**	1.29 ± 0.58	1.42 ± 0.68	1.20 ± 0.50^§^
**FEV_1 _% predicted**	47.7 ± 18.5	53.7 ± 18.6	44.0 ± 17.6^┼^
**Charlson's comorbidity index score**	1 [0-2]	1 [0-2]	1 [0-2]
**Current smoking**	28 (23.1)	8 (17.4)	20 (27.0)
**Recruitment**			
**Scheduled outpatient clinic visit**	83 (86.6)	30 (36.1)	53 (63.9)
**Scheduled general physician visit**	14 (11.6)	5 (35.7)	9 (64.3)
**Discharge from hospital admission**	24 (19.8)	11 (45.8)	13 (54.2)
**Hospital admissions 1yr prior to study**			
**-0**	68 (56.2)	25 (54.3)	43 (57.3)
**-1**	32 (26.4)	15 (32.6)	17 (22.7)
**-2 or more**	21 (17.4)	6 (13.1)	15 (20.0)
**Home oxygen therapy**	12 (9.9)	5 (10.9)	7 (9.3)

Data are expressed as mean ± SD, median [IQR] or count (percentage). ^§ ^p < 0.05; ^┼^p < 0.01.

During the observational period 75 first exacerbations were observed of which 26 (35%) were severity type 1, 20 (27%) type 2, 19 (25.%) type 3, and 10 (13.%) exacerbations were defined as type 4. At exacerbation onset, most patients reported an increase in dyspnea (81%) and increased cough (51%). Furthermore, 27% of exacerbating patients reported increased amounts of sputum and 28% reported a change in sputum purulence.

### Symptomatic days

We analyzed 979 symptomatic days (not part of an exacerbation and preceded by at least three consecutive stable days). A total of 926 (95%) symptomatic days had an increase in one or more minor symptoms, from which a majority of 874 (89%) days were not accompanied by an increase in one or more major symptoms. A total of 86 (9%) symptomatic days had an increase in one major symptom, 14 (1,4%) had an increase in two major symptoms, and 1 (0.1%) had an increase in three major symptoms. No data on action measures was available on 4 (0.4%) days.

### Actions taken by patients

**Figure 2:**
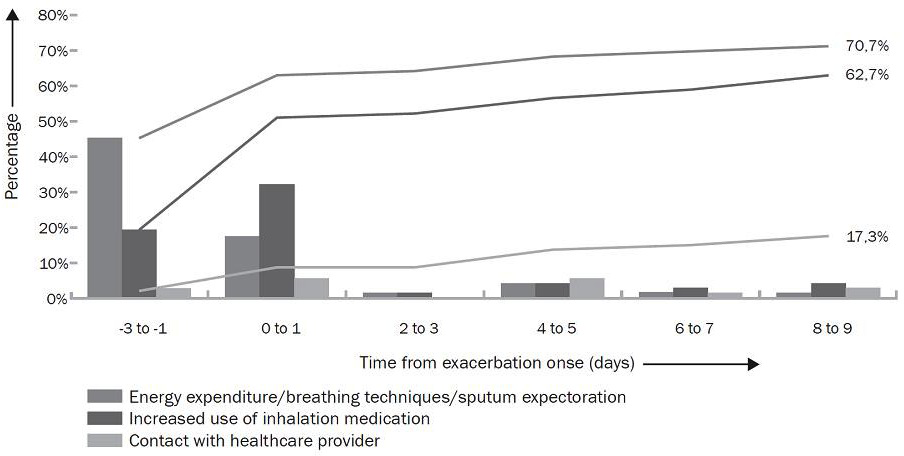
**Incidence and cumulative incidence of three type of action measures performed in the event of an exacerbation**.

Figure 2 shows the incidence and cumulative incidence and timing of actions initiated between three days before to ten days after exacerbation onset. The majority of patients experiencing an exacerbation performed type-A (70.7%) and type-B actions (62.7%), while only 17.3% of the exacerbations were followed by type-C actions. Type-A actions were mainly taken in the days prior to onset (median -3, IQR -3 to 0), while type-B actions were taken at onset (median 0 IQR -1 to 1). Timing of the 17.3% of patients taking type-C actions did not show a clear pattern (median 4 IQR 0.5 to 5.5) in proportion to exacerbation onset.

Table 2 shows patient-, and exacerbation characteristics for the different actions taken. Taking type-A actions was significantly more often performed by non-smoking patients (20.8% vs 42.9%, p = 0.04). Patients taking type A actions had significantly longer duration of exacerbations; 10.4 (95%CI 8.7-12.2) vs 6.5 (95%CI 3.6-8.6) days, p = 0.04). Smokers were also less likely to take type-B actions (17.4% vs 43.9%, p = 0.02). Initiation of type-B actions was significantly associated with dyspnea at onset of the exacerbation (89.4% vs 67.9%, p = 0.02). The opposite was seen when exacerbation symptoms at onset included increased sputum purulence (19.1% vs 42.9%, p = 0.03). Taking type-C actions was significantly associated with more severe airflow obstruction; 35.5 (95%CI 27.4-40.8) vs 45.8 (95%CI 41.4-50.2) %pred, p = 0.04) and increased mean number of hospital admissions in the previous year; 1.9 (95%CI 1.5-2.3) vs 0,6 (95%CI 0.4-0.8), p = 0.001).

**Table 2:** Characteristics of patients taking or not taking timely action measures

		Planning periods of rest, breathing techniques, and sputum expectoration			Increased inhalation medication			Contact with healthcare provider		
		yes	no	p	yes	no	p	yes	no	p
	**N**	53 (70.7)	22 (29.3)		47 (62.7)	28 (37.3)		13 (17.3)	62 (82.7)	
**Patient** **characteristics**	**Sex, male**	34 (64.2)	13 (59.1)	0.68	28 (59.6)	19 (67.9)	0.47	10 (76.9)	37 (59.7)	0.24
	**Age**	66.8 (66.1-71.5)	66.2 (62.1-70.3)	0.80	67.4 (67.4-70.3)	65.4 (61.8-69.0)	0.39	63.0 (57.2-68.8)	67.4 (64.9-69.8)	0.15
	**Living alone**	35 (66.0)	14 (67)	0.96	32 (68.1)	17 (63)	0.65	6 (46)	43 (71)	0.09
	**FEV1-%pred**	41.7 (38.4-44.0)	49.8 (41.5-58.1)	0.07	43.3 (38.0-48.6)	45.3 (39.4-51.2)	0.63	35.5 (27.5-40.8)	45.8 (41.4-50.2)	0.04*
	**Current smoking**	11 (20.8)	9 (42.9)	0.04*	8 (17.4)	12 (43.9)	0.02	2 (15.4)	17 (27.9)	0.35
	**Hospital admissions in previous year**	1.0 (0.6-1.4)	0.4 (0.1-0.7)	0.07	1.0 (0.6-1.4)	0.5 (0.2-0.8)	0.09	1.9 (1.5-2.3)	0.6 (0.4-0.8)	0.001**
	**Charlson's comorbidity score**	1 0 [1,2]	1 0 [1,2]	0.85	1 0 [1,2]	1 [0-1.8]	0.42	1 [0-2.5]	1 0 [1,2]	0.99
**Event** **characteristics**	**Episode length, days**	10.4 (8.7-12.2)	6.5 (3.6-8.6)	0.04*	10.2 (8.0-12.4)	7.8 (5.1-10.5)	0.20	11.6 (7.5 -15.7)	8.8 (6.9-10.7)	0.22
	**Severity**			0.32			0.31			0.42
	**- Anth. I** **- Anth. II** **- Anth. III** **- Anth. IV**	21 (39.6) 15 (28.3) 11 (20.8) 6 (11.3)	5 (22.7) 5 (22.7) 8(36.4) 4 (7.6)		16 (34.0) 10 (21.3) 15 (31.9) 6(12.8)	10 (35.7) 10 (35.7) 4(14.3) 4(14.3)		6 (46.1) 4(30.8) 3(23.1) 0(0.0)	20 (32.3) 16 (25.8) 16 (25.8) 10 (16.1)	
	**Symptom count at onset**	2.7 (2.3-3.1)	2.2 (1.7-2.7)	0.16	2.5 (2.1-2.9)	2.6 (2.1-3.2)	0.72	2.7 (2.0-3.4)	2.5 (2.2-2.9)	0.66
	**Major symptoms at day 1**									
	**- dyspnea** **- sput purulence** **- sput volume**	43 (81.1) 17 (32.1) 15 (28.3)	18 (81.8) 4 (18.2) 5 (22.7)	0.95 0.22 0.62	42 (89.4) 9 (19.1) 10 (21.3)	19 (67.9) 12 (42.9) 10 (35.7)	0.02† 0.03† 0.17	10 (76.9) 5 (38.5) 3(23.1)	51 (82.3) 16 (25.8) 17 (27.4)	0.65 0.35 0.75

Data are expressed as Mean (95%CI), Median [Interquartile range] or count (percentage).

FEV1-% pred: Forced Expiratory Volume in 1 second-percentage of the predicted value; Anth: Anthonissen;

* p ≤ 0.05 Student T-test, ** p ≤ 0.01 Student T-Test, † p ≤ 0.05 Chi-square test

### Actions on symptomatic days

**Figure 3:**
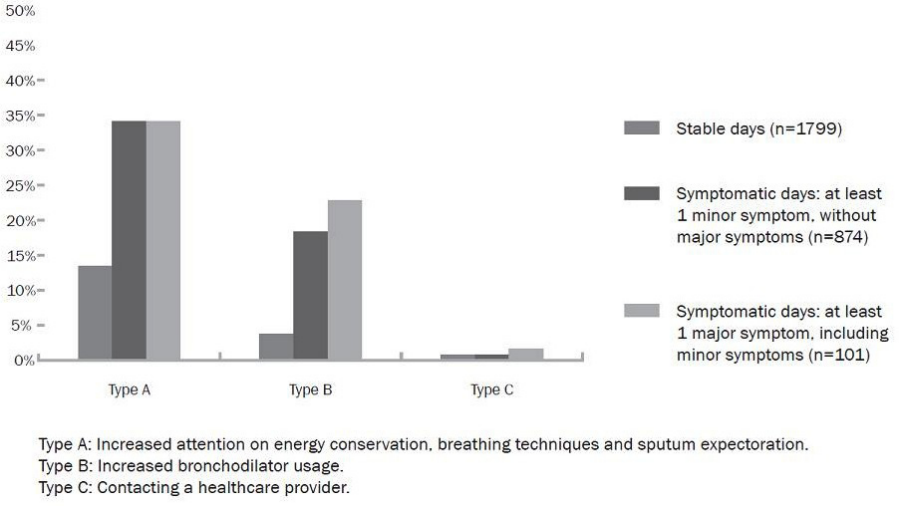
**Actions performed on symptomatic days not part of an exacerbations episode**.

Figure 3 shows actions taken on symptomatic days, not part of an exacerbation. From the 874 symptomatic days with at least one minor symptom, 376 (43.0%) were associated with type-A actions, 201 (23.0%) with type-B and 12 (1.4%) with type-C actions. For symptomatic days with an increase in at least one major symptom (n = 101), these numbers are respectively 43 (42.6%), 29 (28.7%) and 2 (2.0%). In a small proportion of stable days, i.e.with no symptom increase, nevertheless type-A actions were initiated (17,1%). In 5,2% and 0,6% of these stable days patients performed type-B and type-C actions respectively.

## Discussion

The results of this study demonstrate that despite the high amount of unreported exacerbations, most COPD patients do recognize periods of symptom deteriorations and are willing to take certain self-management actions. To our knowledge, this is the first prospective study investigating patient's decisions and self-management behaviour during symptomatic days and exacerbation episodes.

It is well established that COPD exacerbations have clear negative impact on disease progression, morbidity, mortality, and HRQoL [3,5,20]. According to current guidelines, exacerbations should be treated with increased bronchodilator therapy, glucocorticosteroids and/or antibiotics [1]. Patient recognition of exacerbation of symptoms and prompt intervention reduces the risk of hospitalisation and is associated with a better quality of life [11,21]. However, in line with our findings different cohort studies have revealed that only a minority of exacerbations in fact are reported and subsequently treated [8-10]. Despite the fact that reported exacerbations have on average the worst outcomes, unreported exacerbations showed in a Canadian and a Chinese cohort to have important and non-negligible impact on annual change in health status [9,10] Until now, only a few studies examined potential determinants of reporting exacerbations. Delay or failure to report exacerbations seems to be associated with a combination of disease, event and patient characteristics. Our study also confirmed that patients with more severe airflow limitation and higher hospitalisation rates in the previous year are more likely to report an exacerbation [9,22]. In a Canadian cohort, a higher number of symptoms at onset and exacerbations with increased cough and sputum quantity were associated with reporting [9]. In addition, other studies show that psychological characteristics may also be considered as potential predictors of seeking medical treatment [10,23,24].

In contrast to the lack of seeking medical attention, patients did seem to recognize consistent warning signs stimulating them to anticipate on these episodes. Rather than contacting a healthcare provider, the majority (70.7%) of patients showed a willingness to increase their attention on planning periods of rest, breathing techniques and/or sputum expectoration. A similar high proportion of patients increased their bronchodilation medication (62.7%). These findings are in line with two multinational interview-based studies investigating patients perceptions of retrospectively self-reported exacerbations [13,19]. These studies also showed that the majority of COPD patients are able to recognize and respond to periods of symptom deterioration. Only one study reported on exacerbation-related actions focused on planning periods of rest (30% rest; 10% lie/sit down;, 10% stay calm/prepare) [19]. Furthermore both studies found that 33% of the patients anticipated by increasing medication [13,19]. It needs to be emphasized that comparisons partly hampers both because of differences in operational definition of an exacerbation and the methods used to assess these self-management actions.

In the current study, both type-A and type-B actions were conducted timely, mainly during prodrome and at exacerbation onset. The latter is consistent with findings of a previous study reporting that half of the exacerbations were related to self-initiated use of rescue medication, mainly during the week before reporting the exacerbation [22]. Obviously, patients cannot predict whether prodromal days with symptom increase turn into actual exacerbations. This was reflected by examining actions during isolated symptomatic days that were not part of an exacerbation. Around 40% of these days were also associated with type-A actions and 25% with type-B actions. This suggests that a substantial amount of patients seem to respond immediately to symptom changes, even if this only concerns increase in minor symptoms.

In addition to reporting of exacerbations, we related patient characteristics for timely taken type-A and type-B action measures during exacerbations. Interestingly for both types of action, current smoking had an increased likelihood of been associated with less appropriate self-management actions. Similar defaults in self-management behaviour by current smokers were seen from previous studies in using appropriately meter dose inhaler technique [25], being adherent to long term nebulizer therapy [26] and attending outpatient pulmonary rehabilitation [27,28]. Furthermore, correct type-A actions were more frequently taken in patients with longer exacerbations episodes. Finally, the type of symptoms showed to influence patients' self-management decisions during exacerbations. Patients perceiving increased dyspnea at exacerbation onset are more likely to take type-B actions that patients without increased dyspnea. Patients with increased sputum purulence are less likely than patients without increased sputum purulence to take action. We believe this study provides valuable new insights in how patient respond to symptom deterioriation. This knowledge can support the development of improved patient information and material to enhance appropriate self-management of exacerbations. However, a few words of caution are needed when interpreting the results. First, although this study was the first to examine self-management decisions prospectively, it comprised a relatively small group of 108 consecutive patients followed up for only 6 weeks resulting in 75 exacerbations. This is equal to an annual rate of 6.0 per patient-year This relatively high event-rate can be explained by the fact that all patients were simultaneously followed-up in the same 6-week winter period in which exacerbations have shown to be ~ 50% more likely than in other seasons [6,29]. Also the relative high proportion of patients included immediately after hospitalisation might have contributed to a higher exacerbation rate.

Secondly, the relatively short period of follow-up only allowed for the evaluation of a single exacerbation per patient. Data from a well-known UK cohort indicates that exacerbations, although not validated in terms of aetiology, are not random events but clustered in time [30]. The fact that we did not evaluate multiple exacerbation episodes per patient, could have resulted in biased and incomplete judgment of relapsed or recurrent exacerbations and subsequently the presence or absence of correct self-management behavior. A longer follow-up would have created the opportunity to judge within-patient variations over time, taking into account clustering of exacerbations. A stable run-in period of at least 4 weeks could have eliminated recurrent exacerbations [30].

Thirdly, another limiting factor is that after dichotomizing episodes by actions carried out, the number of events per predictor variable did not reach the rule of thumb to allow for multivariate logistic regression analysis [31]. Therefore, the present study solely evaluated the association between single characteristics and actions taken. Adequately powered observational studies allowing multivariate analysis including adjustment for potential confounders are needed to validate current indicators of self-management behaviour.

This study was not designed and powered to examine the effects of different self-management decisions taken by patients on exacerbation related outcome (recovery time, severity etc). Nevertheless, indirectly from the literature, it can be expected that early and self-initiated response on these episodes including all three type of actions most likely affect outcome. Self-management measures during periods of symptom deterioration like changing activity, relaxation, and breathing pattern alteration, a proxy for type-A actions, have shown to be effective in faster symptom relief [32,33]. Furthermore, there is sufficient evidence that prompt anticipation by increasing short-acting bronchodilators (type-B actions) is effective in reducing symptoms and improve airflow obstruction during exacerbations [1,34]. Our study indicates that a substantial amount of exacerbations remain partly or completely unmanaged. As recommended, these results stress the importance of developing and investigating individualized therapies specifically aiming at early recognition and consequent actions to these episodes by patients [35]. Within these interventions, our data suggests that specific attention concerning exacerbation-related health behavior should go out to current smokers.

## Conclusion

This study provides new and important data on decision-making and self-management behaviour during periods of symptom deterioration and exacerbations in patients with COPD. It shows that the majority of patients with COPD are willing to respond promptly when confronted with these acute episodes, but generally refrain from reporting to a healthcare provider. These findings have increased the understanding of patient's perception of exacerbations and predisposal factors of poor self-management. Furthermore, it contributes to the development and effective targeting of exacerbation related self-management interventions.

## List of abbreviations

COPD: Chronic Obstructive Pulmonary Disease; FEV_1_: Forced Expiratory Volume in one second; HRQoL: Health Related Quality of Life; IQR-Inter Quartile Range.

## Competing interests

The authors declare that they have no competing interests.

## Authors' contributions

All authors read and approved the final manuscript. JCAT-leading of development of the study conceptualisation, design, refining of protocol and write up for publication. DS-contribution to refining and substantial contribution to writing up of the protocol for publication. EM-contribution to development of study design, statistical issues, contribution to protocol publication GHWO-major input into study conceptualisation, design and protocol publication. JB-expert respiratory input contribution to study conceptualisation and refining on outcome measures, statistical issues and substantial contribution to writing up of the protocol for publication. TJMV/JWJL/AJPS-contribution to study and intervention development and contribution to protocol publication.

## Pre-publication history

The pre-publication history for this paper can be accessed here:

http://www.biomedcentral.com/1471-2466/11/43/prepub
